# PET Imaging of Post-infarct Myocardial Inflammation

**DOI:** 10.1007/s11886-021-01529-9

**Published:** 2021-07-01

**Authors:** Andrej Ćorović, Meritxell Nus, Ziad Mallat, James H. F. Rudd, Jason M. Tarkin

**Affiliations:** Division of Cardiovascular Medicine, University of Cambridge, Cambridge, UK

**Keywords:** PET, Myocardial infarction, Inflammation, Molecular imaging, Non-invasive imaging, Heart failure

## Abstract

**Purpose of Review:**

To examine the use of positron emission tomography (PET) for imaging post-infarct myocardial inflammation and repair.

**Recent Findings:**

Dysregulated immune responses after myocardial infarction are associated with adverse cardiac remodelling and an increased likelihood of ischaemic heart failure. PET imaging utilising novel tracers can be applied to visualise different components of the post-infarction inflammatory and repair processes. This approach could offer unique pathophysiological insights that could prove useful for the identification and risk-stratification of individuals who would ultimately benefit most from emerging immune-modulating therapies. PET imaging could also bridge the clinical translational gap as a surrogate measure of drug efficacy in early-stage clinical trials in patients with myocardial infarction. The use of hybrid PET/MR imaging, in particular, offers the additional advantage of simultaneous in vivo molecular imaging and detailed assessment of myocardial function, viability and tissue characterisation.

**Summary:**

Further research is needed to realise the true clinical translational value of PET imaging after myocardial infarction.

## Introduction

Myocardial infarction is a major global cause of morbidity and mortality, both due to acute complications arising at the time of initial presentation, as well as longer-term sequelae including chronic heart failure. After myocardial infarction, inflammation and its resolution play key roles in the promotion of healing responses and ultimately act to limit the degree of acute cardiac injury and the severity of subsequent adverse cardiac remodelling.

Indeed, inflammation is an automatic and necessary by-product of ischaemic myocyte necrosis, which seeks to clear and organise the damaged tissue. A broadly biphasic immune response to myocardial infarction occurs, wherein an initial ‘pro-inflammatory phase,’ characterised by the sequential mobilisation of various pro-inflammatory cell types, is followed by a ‘reparative phase’ where the main role of the immune system is to facilitate wound healing, involving myofibroblast proliferation, neovascularisation, fibrosis and scar formation [1]. However, an excessive or unbalanced inflammatory response is associated with an increased risk of long-term heart failure [2].

In the light of the landmark CANTOS and COLCOT trials, which established the proof-of-principle that certain anti-inflammatory therapies can reduce cardiovascular events in patients with prior myocardial infarction and initial data indicating an additional signal for reduction in heart failure–related events, the tantalising prospect is thus presented that pharmacological manipulation of immune responses in the post-infarct period could be clinically beneficial [3, 4, 5••].

Given the variation in clinical presentations and the heterogeneity of inflammatory cell phenotypes at play within the infarcted heart, it is likely that targeted immunosuppressive therapies applied in specific high-risk patient populations would yield far greater overall benefit than more ‘blanket’ broad-spectrum approaches that have been trialled in the past [6].

In this regard, positron emission tomography (PET) imaging presents a unique opportunity to probe and visualise molecular mechanisms occurring after myocardial infarction in vivo*,* and to directly investigate the effects of novel therapies on myocardial inflammation. This article provides a focused review of recent progress in the use of PET imaging for imaging post-infarct myocardial inflammation and related processes.

## Cellular Biology of Post-infarct Inflammation

An understanding of the cellular biology of post-infarct inflammation is important for contextualising the significance of cardiac PET signals, although detailed consideration of this topic is beyond the scope of this article. The reader is directed to more comprehensive reviews elsewhere [1, 7, 8].

The cardiomyocyte necrosis that follows cardiac ischaemia sets in motion a chain of events closely linked to immune activation and resolution, which ultimately results in the formation of stable fibrotic scar tissue. However, if this balanced and coordinated immune response fails, uncontrolled initial or resurgent inflammation, fuelled by dysregulation of the mononuclear phagocytic network, can contribute to the risk of developing long-term heart failure [9].

In the initial pro-inflammatory phase, neutrophils are among the first leucocytes to migrate to the site of injury whereupon they phagocytose cellular debris, degrade extra-cellular matrix via the release of matrix metalloproteinases and generate reactive oxygen species. Monocytes are also recruited from the splenic reservoir and bone marrow via adrenergic signalling, whereupon they differentiate into tissue macrophages and likewise undertake phagocytosis, proteolysis and promote inflammation through generation of reactive oxygen species and secretion of pro-inflammatory cytokines (e.g. IL-1β, IL-6 and TNFα). Monocyte recruitment occurs as early as 30 min after the onset of ischaemia and up to 40% of the monocytes recruited within the first 24 h originate from the spleen [10, 11]. B and T lymphocytes and natural killer cells are also crucial components of the initial and subsequent inflammatory responses. In particular, T regulatory cells help direct the transition from post-infarct inflammation towards resolution [12].

In mouse models, flow cytometry performed after coronary ligation has shown that pro-inflammatory Ly-6C^hi^ monocytes (analogous to classical CD14^+^CD16^−^ and intermediate CD14^+^CD16^+^ monocytes in humans), which have a life span of about 20 h, predominate at days 1–4 post-infarction [13]. By day 5–14 post-infarction, through a combination of differentiation and switch from CCR2 to CXC3CL1-mediated recruitment, these cells are replaced by a predominance of Ly-6C^lo^ monocytes (analogous to non-classical CD14^int^CD16^+^ monocytes in humans), which give rise to macrophages whose secretory cytokine profile includes IL-10, TGFβ and VEGF. These cytokines exert anti-inflammatory actions including downregulation of Th1 cell responses, and promotion of angiogenesis and fibrosis through interactions with endothelial cells and fibroblasts, respectively. This well-characterised transition in monocyte phenotype was also observed in a human post-mortem study [14]. This study showed increased pro-inflammatory CD14^+^CD16^−^ cells in the infarct border zone during the acute inflammatory phase, and comparable numbers of CD14^+^CD16^−^ and non-classical CD14^+^CD16^+^ cells in the infarct core at later time points, which coincided with depletion of splenic monocytes. Although several studies have shown different correlations with peripheral blood monocytes in patients with myocardial infarction, the role of non-classical monocytes in this context remains largely unknown.

In another study of 36 patients, increased peak levels of peripheral CD14^+^CD16^−^ monocytes measured after acute myocardial infarction were negatively associated with the likelihood of longer-term left ventricular functional recovery [15]. Increased leukocyte concentration was also associated with a greater incidence of heart failure hospitalisations in a study of 16,940 men from the general population without prior history of myocardial infarction or stroke enrolled in a cardiovascular screening programme in Sweden [16]. In mice, attenuation of monocyte recruitment to the ischaemic myocardium achieved by B cell depletion and silencing of endothelial cell adhesion molecules or the monocyte chemokine receptor CCR2 were, conversely, associated with preserved ejection fraction post-infarct [17–19]. The beneficial actions of angiotensin-converting enzyme inhibition on left ventricular function after myocardial infarction may also in part be related to reduction in recruitment of splenic-derived monocytes to the healing myocardium [20].

Divergent subsets of cardiac CCR2^+^ and CCR2^−^ macrophages have also been described [21]. While monocyte-derived cardiac CCR2^+^ macrophages are pro-inflammatory, tissue-resident CCR2^−^ macrophages have non-redundant cardioprotective roles in tissue remodelling and cardiac regeneration, and largely self-renew via local proliferation [22]. During post-myocardial infarction remodelling, increases in cardiac macrophages occurring in the remote myocardium are due to local proliferation and to a lesser extent monocyte recruitment [18].

Lower numbers of CCR2^+^ macrophages have been associated with improvement in left ventricular systolic function in patients with severe heart failure undergoing left ventricular assist device implantation, as has also been shown in mice [23, 24]. In other murine studies, directing cardiac macrophage populations towards a reparative phenotype using either small interfering RNA directed at the transcription factor IRF5, or subcutaneous IL-10 injection, led to improvements in left ventricular function post infarct [25, 26].

Traditionally, the functions of the broadly divergent macrophage types in the post-infarct myocardium have been ascribed to ‘M1-like’ and ‘M2-like’ macrophages. However, it is important to note that this classification system is based on a spectrum of in vitro activation profiles rather than the broader transcriptional repertoire of macrophages that is now known to exist [27]. There is also incomplete overlap with M1 and M2 markers in cardiac CCR2^+^/CCR2^−^ macrophages [23]. Indeed, data from studies that have applied unbiased high-throughput sequencing techniques have revealed a wide-range of both resident and monocyte-derived macrophage sub-types that co-exist in the infarct, peri-infarct and remote myocardium [28]. The occurrence of macrophage plasticity and the role of metabolic and epigenetic reprogramming of multi-level systemic immune cell activation add to the level of complexity [29].

Apoptosis and the clearance of apoptotic cells (efferocytosis) are another important component of post-infarction damage resolution, and experimental suppression of efferocytosis has been shown to result in larger myocardial infarct size and greater systolic dysfunction [30].

Because these key inflammatory processes are vital for determining the extent of myocardial damage and likelihood of long-term heart failure after myocardial infarction, there is a role for molecular imaging techniques such as PET to study these disease mechanisms in efforts to inform the design and use of new therapeutic strategies for ischaemic heart failure.

## PET Tracers for Imaging Post-infarct Inflammation

Molecular imaging of myocardial inflammation with PET offers the advantage of enabling the in situ visualisation, in real-time, of post-infarction processes. Moreover, by utilising novel PET tracers that are specific for particular inflammatory cell types or processes, individual components of the initial inflammatory and subsequent healing responses can potentially be interrogated.

### ^18^F-FDG Imaging After Myocardial Infarction

^18^F-fluorodeoxyglucose (^18^F-FDG) is the most commonly used PET tracer for inflammation imaging in clinical medicine, and several studies of post-infarct imaging testify to the general feasibility of this imaging approach.

Using PET/CT in a mouse model of myocardial infarction, one study showed increased ^18^F-FDG uptake within infarcted myocardium on day 5 post-infarct compared to control animals, which co-localised with delayed gadolinium enhancement on MRI [31]. The highest ^18^F-FDG signals were observed at the infarct border zone, which corresponded with the site of peak monocyte/macrophage recruitment in some cases. Increased tracer uptake was also confirmed in monocytes/macrophages isolated from the infarcted mouse hearts, compared to the surrounding infarct tissue.

Similar findings for ^18^F-FDG have been observed in several clinical studies. For example, one study including 15 patients imaged within 7 days of a first myocardial infarction showed the metabolic rate of ^18^F-FDG uptake to be increased in infarcted versus remote myocardium [32]. The PET signal was higher in myocardial segments with late gadolinium enhancement and oedema, versus oedema alone in this study. In 5 control patients with previous stable myocardial infarction, a reverse pattern was observed, with lower ^18^F-FDG uptake in the chronic infarct compared to remote zone. Another study that analysed 39 patients with ST-segment elevation myocardial infarction found that elevated infarct-related ^18^F-FDG uptake in the first week after myocardial infarction was associated with an increased likelihood of left ventricular impairment on MRI after 6 months, independent of infarct size [33•]. In this study, ^18^F-FDG uptake in the infarcted myocardium was also correlated with ^99m^Tc-sestamibi SPECT-defined area at risk and peripheral blood counts of CD14^high^CD16^+^ monocytes.

However, despite these encouraging data, myocardial imaging using ^18^F-FDG is nonetheless challenging due to avid physiological background uptake. Pre-scan patient preparation for cardiac ^18^F-FDG imaging requires cumbersome myocardial suppression protocols, which are often ineffective. The effect of dietary manipulation on the already altered metabolic state of viable, post-ischaemic cardiac myocytes is also unknown. Moreover, while ^18^F-FDG is a clinically useful measure of metabolic activity in inflammatory cells such as macrophages in several cardiovascular diseases, its uptake is not cell-specific.

**Fig. 1:**
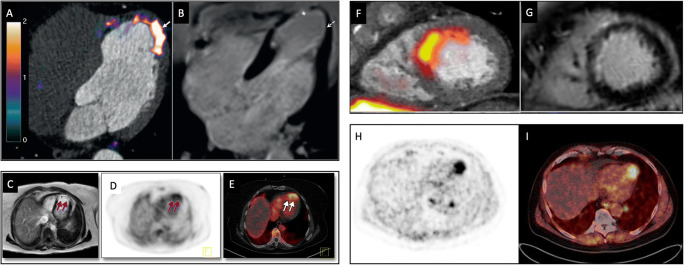
PET imaging of inflammation and related processes after myocardial infarction. ***Left panels***: tracers for imaging inflammatory cells. *Top* **A** ^68^Ga-DOTATATE (SST_2_) PET-CT image demonstrating residual inflammation (*arrow*) in **B** partially viable myocardium with subendocardial infarct (*dashed arrow*), bordering full thickness scarring (*asterisk*) confirmed by late gadolinium enhancement magnetic resonance imaging, 4 years after a left anterior descending artery MI. (Reproduced from: *J Am Coll Cardiol*. 2019;73:2489–91; doi: 10.1016/j.jacc.2019.02.052; Creative Commons user license https://creativecommons.org/licenses/by/4.0/) [34]. *Bottom* An apical myocardial infarct *(arrows*), visualised using contrast-enhanced multishot inversion recovery turbo field echo cardiac MRI (**C**), ^68^Ga-pentixafor (CXCR4) PET (**D**) and fused PET-CT (**E**). (Reprinted from *JACC Cardiovasc Imaging*. 2015;8:1466–8, with permission from Elsevier) [35]. ***Right panels****:* tracers for imaging post-infarct myocardial processes related to inflammation. *Top* ^18^F-Fluciclatide (angiogenesis) PET-CT (**F**) and MRI images (**G**) of an antero-septal infarct. (Reproduced from: *Heart.* 2016;103:607–15; doi: 10.1136/heartjnl-2016-310115; Creative Commons user license https://creativecommons.org/licenses/by/4.0/) [36•]. *Bottom* ^68^Ga-FAPI (fibrosis) imaging. In this patient with thyroid cancer, whole-body PET-CT imaging reveals ^68^Ga-FAPI uptake in the left ventricle as shown on the PET (**H**) and fused PET-CT (**I**) images. (Reproduced from: *J Nucl Cardiol.* 2020;1–10; doi: 10.1007/s12350-020-02307-w; Creative Commons user license https://creativecommons.org/licenses/by/4.0/) [37]

To overcome these limitations, several novel PET tracers that could potentially provide more specific markers of inflammation and/or related cellular processes than ^18^F-FDG have been investigated for use in myocardial imaging (Fig. 1 and Table 1).

**Table 1:** PET tracers for imaging post-infarction inflammation and repair processes, and their targets

Tracer	Molecular target	Cellular target	Biological process
Tracers for imaging post-infarct inflammation			
^18^F-GE180 [38•]	TSPO	Monocytes/macrophages and other inflammatory cells	Inflammatory cell recruitment/activity
^68^Ga-DOTA-TOC [39] ^68^Ga-DOTATATE [34]	SST_2_ receptors	Predominantly ‘pro-inflammatory’ M1 macrophages	Inflammatory cell recruitment/activity
^68^Ga-pentixafor [35, 40, 41, 42•]	CXCR4	Several cell types, including macrophages and T lymphocytes	Inflammatory cell recruitment/activity
^64^Cu-DOTA-ECL1i [43] ^68^Ga-DOTA-ECL1i [44]	CCR2	Monocytes/macrophages	Inflammatory cell recruitment/activity
^64^Cu-Macrin [45•] ^18^F-Macroflor [46]	n/a (Phagocytosed by macrophages)	Macrophages	Inflammatory cell recruitment/activity
^11^C-methionine [47, 48] ^14^C-methionine [49]	n/a (Amino acid analogue)	Macrophages	Inflammatory cell recruitment/activity
Tracers for imaging post-infarct myocardial processes related to inflammation			
^18^F-Galacto-RGD [50] ^68^Ga-PRGD2 [51] ^18^F-Fluciclatide [36•]	Integrin a_v_β_3_	Endothelial cells	Angiogenesis
^68^Ga-FAPI-04 [37, 52, 53]	Fibroblast activation protein 4	Fibroblasts	Fibrosis
^18^F-ML-10 [54] ^18^F-ML-8 [54]	Cell membrane fragments	Multiple cell types	Cellular apoptosis
^18^F-NaF [55]	Hydroxyapatite	n/a	Microcalcification
^68^Ga-NODAGA-exendin-4 [56]	GLP-1R	Macrophages and smooth muscle cells	GLP-1R signalling, which has been linked to attenuated inflammatory responses and fibrosis

(Adapted from*: Curr Cardiol Rep* 22, 119 (2020). 10.1007/s11886-020-01372-4; Creative Commons user license https://creativecommons.org/licenses/by/4.0/) [57]

### Tracers for Imaging Post-infarct Inflammation

Aside from ^18^F-FDG, other PET tracers that have been investigated for post-infarct myocardial inflammation imaging either bind cell-surface receptors that are known to be expressed on macrophages and other inflammatory cells, or are more selectively taken up by metabolically active inflammatory cells than ^18^F-FDG. Importantly, these tracers exhibit lower background myocardial activity than ^18^F-FDG, offering the potential for better differentiation of focal myocardial signals without the need for dietary myocardial suppression. While much of the key research in the field has so far been conducted in preclinical animal models, a summary of novel PET tracers applied in clinical studies is shown in Table 2.

**Table 2:** A summary of key findings from clinical post-infarct PET imaging studies

References	Tracer	Summary of main findings
[32, 33•]	^18^F-FDG	• ^18^F-FDG uptake was significantly increased in infarct versus remote myocardium. • Myocardial PET signals correlated with spleen and bone marrow ^18^F-FDG uptake. • There was an inverse correlation between ^18^F-FDG uptake at baseline and LV function at follow-up, independent of infarct size. • Peripheral blood counts of CD14^high^/CD16^+^ monocytes have been shown to correlate with ^18^F-FDG signals.
[38•]	^18^F-GE180 (TSPO)	• Elevated myocardial TSPO signal was identified in the hypoperfused infarct region at 4 to 6 days after STEMI. • Infarct patients also had higher brain TSPO uptake relative to healthy controls, most pronounced in temporal and frontobasal cortex, hypothalamus and cerebellum
[34, 39]	^68^Ga-DOTA-TOC, ^68^Ga-DOTATATE (SST_2_)	• For ^68^Ga-DOTA-TOC imaging, there was good concordance between PET and cMRI for infarct-positive segments. • ^68^Ga-DOTATATE signals were higher in infarcted versus non-infarcted myocardium. • There was very low background signal for ^68^Ga-DOTATATE compared with ^18^F-FDG PET. • Bone marrow ^68^Ga-DOTATATE signals were highly correlated with myocardial signals as well as with bone marrow ^18^F-FDG activity. • In some cases, ^68^Ga-DOTATATE signals remained elevated in the infarcted territory for several years after the index event.
[35, 40, 41, 42•]	^68^Ga-pentifaxor (CXCR4)	• Tracer uptake was significantly higher in infarct versus remote myocardium and was highest in segments with late enhancement and oedema. • Myocardial signals were paralleled by elevated bone marrow uptake. • Patients have been shown to have variable signal positivity after myocardial infarction. • There was good concordance between PET and cMRI for infarct-positive segments. • In one study of patients imaged within 2–13 days post-symptom onset, CXCR4 PET signals *inversely* correlated with scar volume on follow-up MRI at 1–14 months. • In another study of 50 patients imaged at 3–5 days after STEMI, infarct CXCR4 SUV inversely correlated with LVEF at follow-up among the 29 patients who returned for follow-up cardiac assessment at 7.0 ± 2.8 months. • Infarct CXCR4 signal intensity has been shown to correlate with peak CK and CRP levels.
[47]	^11^C-Methionine	• There was increased ^11^C-Methionine uptake in the infarcted area, versus decreased ^18^F-FDG uptake. • The highest accumulation of ^11^C-Methionine was observed in the early phase after MI and was greatest in the infarct border zone. • In the 2 patients with longer-interval follow-up scans, ^11^C-Methionine signals declined over time and were almost undetectable at 6 months.
[36•, 51]	^68^Ga-PRGD2, ^18^F-Fluciclatide (Integrin a_v_β_3_)	• Patchy ^68^Ga-PRGD2 uptake occurred in or around the ischaemic regions in 20/23 MI patients. • Higher uptake was observed at 1–3 weeks after the initial event. • ^68^Ga-PRGD2 uptake positively correlated with size of infarct. • Smaller or older lesions displayed no ^68^Ga-PRGD2 uptake. • ^18^F-Fluciclatide uptake was increased in acutely infarcted versus remote myocardium and compared with uptake in healthy volunteers. • There was no ^18^F-Fluciclatide uptake at sites of established prior infarction in patients with CTO. • ^18^F-Fluciclatide uptake was increased in segments displaying functional recovery on follow-up cMRI.
[37, 53]	^68^Ga-FAPI-04	• In retrospective studies, myocardial tracer uptake correlated with coronary artery disease, age and LVEF and/or other cardiovascular risk factors.
[55]	^18^F-NaF	• ^18^F-NaF tissue-to-background ratios were higher in infarcted versus remote myocardium.

#### Translocator Protein Receptor

The translocator protein (TSPO) receptor is situated in the outer mitochondrial membrane and is expressed in activated mononuclear cells and other inflammatory cells. In a translational study, PET imaging with the TSPO tracer ^18^F-GE180 revealed elevated myocardial TSPO signals at 1 week post-coronary artery ligation in mice, which co-localised to CD68^+^ inflammatory macrophages in the infarct [38•]. Early increases in TSPO PET signals after the infarct were predictive of subsequent adverse left ventricular remodelling at 8 weeks. In vitro data also demonstrated that M1 macrophages exhibited 7-fold higher TSPO uptake compared to M2 macrophages. Treatment of mice with an angiotensin-converting enzyme inhibitor also resulted in lower TSPO signal in the infarct compared to control animals. In the same study, feasibility of clinical imaging was shown in 3 patients with myocardial infarction who were found to have elevated infarct-related TSPO signal compared with healthy controls. Interestingly, changes in neuroinflammation detected by TSPO occurred in parallel to changes in infarct-related myocardial signals in both mice and patients.

#### Somatostatin Receptor Subtype-2

Upregulation of somatostatin receptor subtype-2 (SST_2_) occurs in activated macrophages, offering another novel inflammation imaging target that may be useful in range of cardiovascular diseases [58, 59]. Among the clinically available somatostatin receptor PET tracers, ^68^Ga-DOTATATE has the highest binding affinity for SST_2_. Although one preclinical study observed rapid blood clearance of ^68^Ga-DOTATATE following injection with no myocardial binding after coronary ligation in mice, initial clinical data appears more promising for this approach. In a study that included 6 patients with myocardial infarction who were imaged using the somatostatin receptor PET tracer ^68^Ga-DOTATOC within 3 to 10 days of symptoms, focal myocardial signals were observed in all patients [39]. Overall, there was good concordance between ^68^Ga-DOTATOC signal and infarcted myocardial segments with late gadolinium enhancement and oedema on MRI. In an exploratory post hoc analysis of 12 patients with myocardial infarction who underwent prospective cardiovascular ^68^Ga-DOTATATE PET imaging, infarct-related myocardial SST_2_ signals were increased in patients with recent myocardial infarction [34] (Fig. 1). In this study, focal myocardial ^68^Ga-DOTATATE signals were observed in damaged myocardial segments with echocardiographic regional wall motion abnormalities in patients with old myocardial infarction. Infarct-related ^68^Ga-DOTATATE signals were also correlated with increased metabolic bone marrow activity measured by ^18^F-FDG PET. A pre-specified sub-study of an ongoing prospective observational study aiming to characterise the natural history of coronary and myocardial ^68^Ga-DOTATATE inflammatory signals after myocardial infarction (ClinicalTrials.gov Identifier: NCT04073810) will shed further light on these initial observations.

#### C-X-C Chemokine Receptor Type-4

The C-X-C motif chemokine receptor type 4 (CXCR4) is expressed on the surface of several inflammatory cell types, including macrophages, neutrophils and lymphocytes. In one study, uptake of the CXCR4-targeted tracer ^68^Ga-pentixafor peaked in the infarct region 3 days post-myocardial infarction in mice and corresponded with a flow cytometry-based peak of circulating CD45^+^ leukocytes and histological staining of macrophages and granulocytes within the infarct [40]. As with the TSPO ligand, CXCR4 PET signals in mouse infarcts were also attenuated by pre-administration of enalapril. As part of the same study, feasibility of clinical imaging was shown in 12 patients who underwent ^68^Ga-pentixafor PET/CT and cardiac MRI within 8 days of an ST-segment elevation myocardial infarction. In these patients, CXCR4 PET signals were higher in the infarct region than remote myocardium and were correlated with CXCR4 signals in the bone marrow and spleen.

In a small clinical study of 7 patients with myocardial infarction who underwent ^68^Ga-pentixafor PET imaging within 5–10 days of the event, only 3 of the 7 patients showed focal uptake in the infarct as defined by MRI [35] (Fig. 1). Another study showed infarct-related ^68^Ga-pentixafor uptake in 17 of 22 patients imaged within the first 2 weeks after the event [41]. In this study, myocardial CXCR4 signals were inversely associated with time from symptoms to scanning and were also negatively associated with scar volume assessed by late gadolinium MRI at a median 4-month follow-up. Residual myocardial ^68^Ga-pentixafor uptake at this late timepoint could represent leucocyte involvement in the healing process, but without direct histological comparison, this notion remains speculative.

In another translational study involving 180 mice, persistent myocardial ^68^Ga-pentixafor PET signals occurring beyond 3 days after coronary ligation were observed more often in animals that died of left ventricular rupture than those who survived and were associated with worse cardiac function at 6 weeks [42•]. Moreover, the incidence of left ventricular rupture was reduced by administration of a CXCR4 blocking agent within the first 3 days post-MI. When compared to histology, myocardial ^68^Ga-pentixafor uptake co-localised with CD68^+^ inflammatory cells and was also associated with increased numbers of both Ly6C^high^ and Ly6C^low^ peripheral monocyte counts. These findings were confirmed in 50 patients with myocardial infarction. In these patients, CXCR4 signals showed a weak-to-moderate inverse correlation with left ventricular function at baseline and after follow-up of approximately 7 months.

Another study showed increased ^68^Ga-pentixafor uptake in mediastinal lymph nodes in patients after myocardial infarction because of T cell accumulation [60]. The findings of this study support a potential role for CXCR4 blockade post-infarction as a means of mobilising T-regulatory cells to the injured myocardium, which have been shown to limit adverse cardiac remodelling in pre-clinical models [61].

#### Chemokine Receptor Type-2

C-C chemokine receptor type 2 (CCR2) is another important inflammatory cell marker involved in monocyte/macrophage recruitment, which has been evaluated for imaging myocardial inflammation using PET. In an ischaemia-reperfusion heterotopic heart transplantation mouse model, the PET tracer ^64^Cu-DOTA-ECL1i was shown to bind CCR2^+^ monocytes and macrophages within donor hearts, demonstrating increased tracer uptake compared to native hearts [43]. Specific autoradiographic binding was also confirmed in human myocardial specimens. Comparable myocardial PET signal intensity was observed with the ^64^Cu-labelled tracer to data from a previous study on ^68^Ga-DOTA-ECL1i [44].

As CCR2^+^ macrophage depletion has demonstrated beneficial effects on cardiac function post-infarction in mouse models, CCR2-targeted PET tracers could also be useful for the clinical evaluation of CCR2 inhibitors in future early-stage clinical trials [24].

#### Macrophage Phagocytosis

^64^Cu-Macrin is a 20-nm spherical dextran nanoparticle tracer designed specifically to target macrophages. In a preclinical study, ^64^Cu-Macrin was shown to report on the accumulation of macrophages within the infarcted myocardium in experiments using mice, rabbits and pigs [45•]. Increases in lung and cardiac ^64^Cu-Macrin PET signals were also observed in mouse models of sepsis and pneumonia, in newborn mice exhibiting higher macrophage content than adults, and in atherosclerotic plaques in rabbits. Comparison of a near-infrared fluorescent version of the nanoparticle with flow cytometry and confocal staining showed that the tracer uptake occurred predominately in MHCII^high^ CCR2^high^ tissue macrophages. In contrast, ^64^Cu-Macrin uptake was negligible in neutrophils, fibroblasts and endothelial cells.

#### Amino Acid Metabolism

Radio-labelled amino acid analogues represent another potential approach for imaging the metabolic activity of inflammatory cells after myocardial infarction. For example, the PET tracer ^11^C-methionine is thought to accumulate in macrophages, but not healthy myocytes. In a small clinical study, ^11^C-methionine PET was performed in 9 patients with left anterior descending artery myocardial infarctions treated by percutaneous coronary intervention, within 2 weeks of the event [47]. In these patients, increased ^11^C-methionine signal was observed in areas of low or absent ^201^Thallium SPECT and ^18^F-FDG PET signal in the myocardium indicating tissue infarction, and subsequent reductions in ^11^C-methionine signals were seen in 2 patients imaged at 3 or 6 months. In another study, ^11^C-methionine PET imaging performed in mice showed increased tracer uptake in the infarcted myocardium at day 3 post-coronary ligation, compared to the remote myocardium and healthy control animals, which was associated with macrophage infiltration on histology [48]. The ^11^C-methionine PET signal declined by day 7 and was also inhibited by treatment with anti-integrin antibodies that lowered macrophage content. In the same study, ^11^C-methionine uptake was shown to occur more in M1 than M2 polarised macrophages. Further spatiotemporal profiling of infarct-related ^14^C-methionine signal was investigated in a rat model, which showed peak tracer uptake occurring at days 3 to 7 post-infarct, with a gradual decline until day 28 [49]. Lower tracer uptake occurring at later time points was felt to reflect myofibroblast activity or neoangiogenesis, rather than acute inflammation *per se*.

### Tracers for Imaging Post-infarct Myocardial Processes Related to Inflammation

#### Neoangiogenesis

Integrin a_v_β_3_ expressed by endothelial cells (as well as macrophages and other cells) has been examined for use as a PET imaging target for detecting neoangiogenesis. In one study, myocardial accumulation of the a_v_β_3_-targeted PET tracer ^18^F-Galacto-RGD was observed from 3 days post-infarction in rats, with peak tracer uptake in the infarct between 1 and 3 weeks [50]. a_v_β_3_ PET signal intensity was associated with increased histological vascular density in post-infarct tissue specimens. In a clinical study, increased myocardial a_v_β_3_ PET signal was also observed using ^68^Ga-PRGD2 in 20 out of 23 patients imaged within 1 week after their event [51]. Another clinical study showed that ^18^F-Fluciclatide, which also binds a_v_β_3_, showed higher PET signals at the site of acute infarction in patients within 2 weeks of their event, compared to remote myocardium and patients with old infarction or healthy volunteers [36•] (Fig. 1). In this study, baseline ^18^F-Fluciclatide uptake was also associated with an increased probability of subsequent functional left ventricular recovery after 9 months.

#### Cardiac Fibrosis

Experimental tracers have been tested for imaging myofibroblast activity after myocardial infarction, which bind fibroblast activation protein. In one study performed in a rat model of infarction, ^68^Ga-FAPI-04 PET signals occurring at day 6 post-infarction were highest at the infarct border zone, which co-localised with histological myofibroblast staining [52]. A retrospective analysis of clinical images from 32 patients who underwent prior ^68^Ga-FAPI PET imaging for cancer staging showed focal myocardial ^68^Ga-FAPI uptake in patients with previous history of coronary artery disease and impaired left ventricular function [37] (Fig. 1). A similar association between focal cardiac ^68^Ga-FAPI PET uptake and cardiovascular risk factors was found in another retrospective study of 229 patients with cancer [53].

#### Apoptosis and Microcalcification

Other pathogenic processes occurring after myocardial infarction that have been targeted for molecular imaging with PET tracers include apoptosis and microcalcification. Indeed, given the importance of efferocytosis on post-infarct repair and inflammation resolution, detection of defective or delayed clearance of apoptotic cells by PET imaging could represent an important imaging target. A study of the novel apoptosis tracers ^18^F-ML-10 and ^18^F-ML-8 demonstrated focal infarct-related myocardial tracer accumulation on days 1 to 3 after coronary ligation in rats in regions devoid of ^18^F-FDG uptake and histologically confirmed apoptosis [54]. In this study, ex vivo binding assays showed that ^18^F-ML-8 was taken up by apoptotic, but not necrotic or normal cells, presenting a potential advantage over Annexin V and synaptotagmin-I targeted SPECT tracers, which bind exposed phosphatidylserine in both apoptotic and necrotic cells.

Microcalcification is also thought to occur as part of the healing process after myocardial infarction. In one study of 10 patients, signal from the PET tracer ^18^F-NaF, which binds to areas of microcalcification and exposed hydroxyapatite in atherosclerosis, was increased more in areas of myocardial infarction with scar than the remote myocardium [55]. However, areas of increased myocardial ^18^F-NaF PET signals occurring 1 day after myocardial infarction in rats were found to closely match histological markers of apoptosis, but not calcification.

#### Other Molecular Targets

Among the other molecular targets that have been examined for post-myocardial infarction PET imaging is glucagon-like peptide-1 (GLP-1) [56]. This target is of particular interest as inhibition of the GLP1 receptor has been associated with improved cardiovascular outcomes in multiple clinical trials of patients with diabetes mellitus, although its effects on myocardial function are less clear [62]. Novel tracers for myocardial perfusion/infarct imaging, such as ^68^Ga-DOTA chelate, and for imaging myocardial oxidative metabolism, such as ^11^C-acetate, have also been investigated in preclinical studies and could be useful in this setting [63, 64].

## Future Directions

Among the potential clinical translational applications of post-infarct myocardial PET imaging are as follows: (i) to identify and risk-stratify patients with maladaptive initial or ‘residual’ inflammatory responses contributing an increased likelihood of long-term heart failure, and (ii) to inform the design and use of emerging immune-modulatory therapies for cardiovascular disease. This type of mechanistic research may also yield valid, non-PET biomarkers that can be more easily rolled out in the clinic.

Several preclinical studies have shown that PET imaging, for example with tracers targeting TSPO or CXCR4, can quantify changes in myocardial inflammation after treatment with conventional heart failure therapies, such as angiotensin-converting enzyme inhibitors and angiotensin receptor blockers [38•, 40, 65, 66]. In another study, treatment with a novel NOD-like receptor protein 3 inflammasome inhibitor MCC950 in mice reduced infarct-related ^18^F-FDG inflammatory signals, along with histological grading of M1 macrophage and neutrophil infiltration, and improved myocardial viability after myocardial infarction [67]. Results of future clinical trials using PET imaging to examine the effects of novel therapies for modulating ischaemic myocardial inflammation are awaited.

Multi-tracer studies can potentially help further tease out individual inflammatory pathways or healing responses to improve the precision of therapeutic targeting after myocardial infarction. For example, a study published as an abstract showed that peripheral macrophage depletion in mice resulted in reduced ^18^F-GE180 infarct-related uptake at day 7 compared to control myocardial infarction due to lower CD68^+^ macrophage content, but increased ^68^Ga-pentixafor signal because of sustained recruitment of Ly6G^+^ neutrophils, as well as active calcification of intracavity thrombus detected by ^18^Na-NaF [68].

Moreover, the use of multi-modality multi-parametric imaging, and in particular hybrid PET/MRI, offers the ability to combine molecular imaging of inflammation and adjunct processes with detailed tissue characterisation. The potential value of this combined approach was shown in a study of 25 patients who underwent hybrid ^18^F-FDG PET/MRI within 5 days of revascularisation for myocardial infarction [69]. This study found that although changes in ^18^F-FDG uptake, T1 mapping and extracellular volume tended to overlap anatomically within the infarcts of individual patients, a lack of quantitative correlation meant that these measures were reporting on different underlying biological processes or tissue properties. In another preclinical study, dual-imaging with ^18^F-Macroflor PET (a dextran nanoparticle similar to ^18^F-Macrin) and MRI with MPO-Gd, which detects myeloperoxidase activity in neutrophils and Ly-6C^high^ monocytes, was performed at day 2 and 6 post-infarct in mice. This study showed divergent temporal changes in PET and MRI signals in keeping with the well-characterised biphasic monocyte/macrophage phenotypic response after myocardial infarction [46].

## Conclusions

Dysregulated post-infarction inflammation and remodelling confer adverse outcomes in patients after myocardial infarction. PET imaging of these processes can potentially be utilised in both the development and deployment of novel immunomodulatory therapies for this indication. Further research efforts should be directed towards realising the full potential of these novel molecular imaging techniques.
